# Developmental changes in intercellular junctions and Kv channels in the intestine of piglets during the suckling and post-weaning periods

**DOI:** 10.1186/s40104-016-0063-2

**Published:** 2016-01-27

**Authors:** Jing Wang, Liming Zeng, Bie Tan, Guangran Li, Bo Huang, Xia Xiong, Fengna Li, Xiangfeng Kong, Gang Liu, Yulong Yin

**Affiliations:** Hunan Provincial Engineering Research Center for Healthy Livestock and Poultry Production; Key Laboratory of Agro-ecological Processes in Subtropical Region, Institute of Subtropical Agriculture, Chinese Academy of Sciences, Changsha, Hunan 410125 China; University of the Chinese Academy of Sciences, Beijing, 10008 China; Science College of Jiangxi Agricultural University, Nanchang, Jiangxi 330045 China; Hunan Collaborative Innovation Center for Utilization of Botanical Functional Ingredients, Hunan Collaborative Innovation Center of Animal Production Safety, Changsha, Hunan 410000 China

**Keywords:** Intercellular junctions, Kv channels, Small intestine, Piglets, Weaning

## Abstract

**Background:**

The intestinal epithelium is an important barrier that depends on a complex mixture of proteins and these proteins comprise different intercellular junctions. The purpose of this study was to investigate the postnatal and developmental changes in morphology, intercellular junctions and voltage-gated potassium (Kv) channels in the intestine of piglets during the suckling and post-weaning periods.

**Results:**

Samples of the small intestine were obtained from 1-, 7-, 14-, and 21-d-old suckling piglets and piglets on d 1, 3, 5, and 7 after weaning at 14 d of age. The results showed that the percentage of proliferating cell nuclear antigen (PCNA)-positive cells and alkaline phosphatase (AKP) activity, as well as the abundances of E-cadherin, occludin, and Kv1.5 mRNA and claudin-1, claudin-3, and occludin protein in the jejunum were increased from d 1 to d 21 during the suckling period (*P* < 0.05). Weaning induced decreases in the percentage of PCNA-positive cells, AKP activity and the abundances of E-cadherin, occludin and zonula occludens (ZO)-1 mRNA or protein in the jejunum on d 1, 3 and 5 post-weaning (*P* < 0.05). There were lower abundances of E-cadherin, occludin and ZO-1 mRNA as well as claudin-1, claudin-3 and ZO-1 protein in the jejunum of weanling piglets than in 21-d-old suckling piglets (*P* < 0.05). The abundances of E-cadherin, occludin, ZO-1 and integrin mRNA were positively related to the percentage of PCNA-positive cells.

**Conclusion:**

Weaning at 14 d of age induced damage to the intestinal morphology and barrier. While there was an adaptive restoration on d 7 post-weaning, the measured values did not return to the pre-weaning levels, which reflected the impairment of intercellular junctions and Kv channels.

## Background

The gastrointestinal tract is lined by single layer of epithelial cells which play crucial roles in digestion and the absorption of nutrients, as well as in the maintenance of a physical and functional barrier [1, 2]. Young animals experience abrupt changes and challenges at birth and weaning. The development and maturation of the intestinal tract is important for these animals to survive these changes and maintain intestinal health [3].

Weaning stress induces marked morphological and functional changes in the small intestine, and generally results in villus atrophy, crypt hyperplasia, increased transepithelial permeability, decreased digestive and absorptive capacity and impaired intestinal barrier [4–7]. Restitution of the intestinal mucosa is the rapid re-establishment of epithelial integrity and continuity, which includes the migration of epithelial cells adjacent to the injured surface into the wound, followed by epithelial cell proliferation, maturation and differentiation [8, 9]. Differentiated intestinal epithelial cells exhibit increased migration after wounding partially through the activation of voltage-gated potassium (Kv) channel expression, which leads to an increase in the driving force for Ca^2+^ influx during restitution [10]. Meanwhile, the integrity of the intestinal epithelial barrier depends on a combination of proteins that constitute multiple intercellular junctions, including tight junctions and adherens junctions [11–13].

The present study was conducted to investigate the developmental changes in the morphological structure, mucosal cell proliferation, intercellular junctions and Kv channels in the small intestine of piglets during the suckling and post-weaning periods, which should help to improve the adaptation to weaning in piglets.

## Methods

The animal experiments were approved by the Institutional Animal Care and Use Committee of the Institute of Subtropical Agriculture, Chinese Academy of Sciences (2013020).

### Animals and experimental design

The animal experimental design was conducted according to the description in the previous study [14]. Sixty-four neonatal piglets (Duroc × Landrace × Large Yorkshire) from 8 litters (8 piglets per litter) were assigned to 8 groups on the basis of different litter origins and similar body weights. All piglets were housed in an environmentally controlled farrowing cage with hard plastic slatted flooring, and had free access to drinking water. To study the postnatal ontogeny morphology in the intestine, piglets were to be nursed by sows until they were 21 d old and samples were to be collected on d 1, 7, 14 and 21 of age. Another group of piglets were weaned at age 14 d and housed in the same farrowing cage without a sow and fed creep feed (Artificial milk 101, Anyou Feed, China); samples were collected on d 1, 3, 5 and 7 post-weaning to investigate the developmental changes in morphology in the intestine. Eight piglets (one from each litter) were slaughtered and weighed at each of the ages shown above; body weights are shown in Table 1. Five mL of blood was collected aseptically in aseptic capped tubes containing sodium heparin from a jugular vein 2 h after feeding, and centrifuged at 2000 × g for 10 min at 4 °C to obtain plasma samples, which were then stored at -80 °C for diamine oxidase (DAO) and D-lactate analysis. After being stunned electrically, piglets were killed and the small intestine was rinsed thoroughly with ice-cold physiological saline. The middle segments of the jejunum (2 cm) and ileum (2 cm) were cut and fixed in 4 % formaldehyde or 2.5 % glutaraldehyde for morphological and immunohistochemical analysis. Samples of the jejunal and ileal mucosa were scraped, immediately snap-frozen in liquid nitrogen and stored at -80 °C for the determination of alkaline phosphatase (AKP) activity and RNA extraction.

**Table 1:** The body weight of suckling and weaned piglets

Items	Day age				Day post-weaning			
	1	7	14	21	1	3	5	7
BW, g	1604	2932	4279	5792	3967	4399	4813	5355

*BW* were obtained from 1, 7, 14, 21-d-old suckling piglets and piglets on days 1, 3, 5, 7 post-weaning at 14 d of age

### Plasma diamine oxidase and D-lactate

The reaction system for determining the plasma concentration of DAO included 0.1 mL (4 μg), horseradish peroxidase solution (Sigma-Aldrich, St. Louis, MO, USA), 3 mL phosphate-buffered saline (PBS) (0.2 mol/L, pH 7.2), 0.1 mL O-dianisidine methanol solution (500 μg of O-dianisidine) (Sigma-Aldrich, St. Louis, MO, USA), 0.5 mL sample and 0.1 mL substrate solution (175 μg of cadaverine dihydrochloride) (Sigma-Aldrich, St. Louis, MO, USA). The processed samples were incubated in an incubator chamber at 37 °C for 30 min and measured at 436 nm by a UV/visible spectrophotometer-UV-2450 (SHIMADZU, Kyoto, Japan) [15]. Plasma D-lactate was determined using a D-lactate Assay Kit (BioVision, Mountain View, CA, USA) in accordance with the manufacturer’s instructions.

### Intestinal morphology

The segments of the jejunum and ileum fixed in 4 % formaldehyde were used to determine morphology using hematoxylin-eosin staining. After dehydration, embedding, sectioning, and staining, images were acquired at various magnifications with computer-assisted microscopy (Micrometrics TM; Nikon ECLIPSE E200, Tokyo, Japan). Villous height, crypt depth, goblet cell and lymphocyte counts were measured by Image-Pro Plus software, Version 6.0 on images at 200- or 400-fold magnification in five randomly selected fields, respectively [4].

Segments of the jejunum and ileum at higher magnification were also designated for analysis by scanning electron microscopy as described by German [16] and Liu et al. [17]. Briefly, tissue segments were fixed with 2.5 % glutaraldehyde for 2 h at 4 °C, and rinsed 3 × 10 min in PBS at 4 °C. The tissues were then postfixed in 1 % osmium tetroxide for 12 h at 4 °C, and rinsed 3 × 10 min in PBS at 4 °C. After dehydration in a graded ethanol series, the tissues stored in tert butyl alcohol for 2 h at room temperature. After samples were mounted onto stubs by means of quick-drying silver paint, the tissues were coated with gold-palladium and examined by a JEOL JSM-6360LV scanning electron microscope at 25 KV. The apparent characteristics of microvillus were observed and described.

### Alkaline phosphatase activity in the jejunal mucosa

Jejunal mucosa samples were homogenized in 10 volumes (w/v) of ice-cold saline and centrifuged at 6,000 g for 20 min at 4 ^o^C, and the supernatant was used for enzyme and protein analysis. AKP activity was assayed by measuring the release of *p*-nitrophenol from *p*-nitrophenyl phosphate using a commercial kit (Beyotime Institute of Biotechnology, Shanghai, China) and normalized to protein content.

### Real-time quantitative RT-PCR

The abundances of E-cadherin, occludin, zonula occludens (ZO)**-**1, integrin, Kv1.1, and Kv1.5 mRNA in jejunal and ileal mucosa were determined by real-time quantitative RT-PCR. Total RNA was isolated from the liquid nitrogen-pulverized jejunal and ileal mucosa samples with the TRIZOL reagent (Invitrogen, Carlsbad, CA, USA) according to the manufacturer’s instructions and quantified by electrophoresis on 1 % agarose gel with the measurement of optical density at 260 and 280 nm. Synthesis of the first strand (cDNA) was performed with 5 × PrimeScript Buffer 2 and PrimeScript reverse transcriptase Enzyme Mix 1 (Takara Biotechnology (Dalian) Co., Ltd, Dalian, China). Primers were designed with Primer 5.0 (PREMIER Biosoft International, Palo Alto, CA, USA) according to the gene sequence of the pig to produce an amplification product, as described in a previous article [18]. Beta-actin was used as a housekeeping gene to normalize target gene transcript levels [19]. The resulting cDNA was diluted and used as a PCR template to evaluate gene expression. The reaction was performed in a volume of 10 μL (ABI Prism 7700 Sequence Detection System; Applied Biosystems, Foster City, CA, USA). Real-time PCR was performed according to a previous study [20]. Briefly, 1 μL cDNA template was added to a total volume of 10 μL containing 5 μL SYBR Green mix, 0.2 μL Rox, 3uL dH_2_O, and 0.4 mol/L each of the forward and reverse primers. The following protocol was used: (i) pre-denaturation program (10 s at 95 ^o^C); (ii) amplification and quantification program, repeated for 40 cycles (5 s at 95 ^o^C, 20 s at 60 ^o^C); and (iii) melting curve program (60-99 ^o^C with a heating rate of 0.1 ^o^C/s and fluorescence measurement). The comparative Ct value method was used to quantify expression levels of target genes relative to those for β-actin. Data are expressed relative to the values in piglets at d 1.

### Immunohistochemical analysis

The protein abundances of proliferating cell nuclear antigen (PCNA) in the jejunum and tight junction proteins (ZO-1, Occludin, Claudin-3, Claudin-1) in the jejunum and ileum of piglets were determined using an immunohistochemical analysis as described by Wang et al. [18]. Briefly, tissue pieces were fixed with 4 % paraformaldehyde and then serial paraffin-embedded sections were made. After being dewaxed in xylene and re-hydrated via a graded series of alcohol, the sections were heated by microwave in 0.01 mol/L citral acid solution for antigen retrieval and blocked with 4.5 % hydrogen peroxide in phosphate-saline buffer for 15 min. The sections were incubated with anti PCNA (1:200; Wuhan Boster Biological Technology Co., Ltd., Wuhan, China), ZO-1 polyclonal antibody (1:100; Abcam, Cambridge, UK), Occludin polyclonal antibody (1:100; Abcam, Cambridge, UK), Claudin-3 polyclonal antibody (1:100; Abcam, Cambridge, UK), or Claudin-1 polyclonal antibody (1:100; Abcam, Cambridge, UK) overnight at 4 °C, respectively, and then washed three times for 5 min in PBS and incubated with an SV Mouse or Rabbit Hypersensitivity Two-step Immunohistochemical Kit (Boster Biological Technology, Wuhan, China) overnight at 4 °C according to the manufacturer’s instructions. The sections were washed three times for 3 min with PBS, followed by the addition of diaminobenzidine (Boster Biological Technology, Wuhan, China) as a chromogen for 3 to 5 min, which was strictly controlled under a microscope. Before staining, the primary antibodies were replaced by PBS as a negative control. After being rinsed under cold tap water for 5 min and counterstained with Hematoxylin (Boster Biological Technology, Wuhan, China), sections were dehydrated through an alcohol gradient and covered by general clarity gum. The stained sections were acquired using a (Olympus, Tokyo, Japan), and scored independently by Image-Pro Plus software, Version 6.0. The PCNA labeling index was expressed as the ratio of cells that were positively stained for PCNA to all epithelial cells and the abundances of tight junctions proteins were expressed as the average optical density (the ratio of integral optical density to the area of tissue) in at least 5 areas that were randomly selected for counting at 400-fold magnification. All data are expressed relative to the values in piglets at d 1.

### Statistical analysis

All statistical analyses were performed by one-way ANOVA using SPSS software 19.0 (SPSS Inc., Chicago, IL, USA). The differences among treatments were evaluated using Tukey’s test. Differences were considered to be significant at *P* < 0.05. Pearson correlation coefficients were evaluated to describe the relationships between the percentage of PCNA-positive cells or the AKP activity and the relative mRNA abundances of E-cadherin, occludin, ZO-1, integrin, Kv1.1 and Kv1.5 in the jejunal mucosa of piglets.

## Results

### Plasma concentrations of diamine oxidase and D-lactate

Intestinal permeability was detected by monitoring the concentrations of plasma DAO and D-lactate. As shown in Table 2, the DAO content in plasma decreased on d 14 and 21 compared that on d 1 and 7, while the plasma concentration of D-lactate increased on postnatal d 7 and then declined until d 21 in suckling piglets (*P* < 0.05). Within the first week post-weaning after 14 d of age, the concentrations of plasma D-lactate on d 3, 5 and 7 were significantly lower than that at age d 14 and there was a lower concentration in weanling piglets than in suckling piglets at d 21 of age (*P* < 0.05), while there were no diffirence in plasma DAO content among weanling piglets on d 1, 3, 5 and 7 post-weaning. The increased D-lactate content on d 1 post-weaning indicated an increased permeability of intestinal tissue.

**Table 2:** Plasma concentrations of diamine oxidase and D-lactate in the suckling and weaned piglets

Items	Day age				Day post-weaning				SEM	*P*-value
	1	7	14	21	1	3	5	7		
Diamine oxidase, mg/mL	55.26^a^	44.84^ab^	40.52^b^	45.47^ab^	48.54^ab^	44.16^b^	45.00^ab^	41.18^b^	1.28	0.004
D-lactate, mmol/mL	14.49^b^	23.42^a^	17.15^ab^	12.91^b^	18.74^a^	6.08^c^	5.60^c^	5.66^c^	1.34	<0.001

Plasma were obtained from 1, 7, 14, 21-d-old suckling piglets and piglets on d 1, 3, 5, 7 post-weaning at 14 d of age

*n =* 8. ^a-c^Values with different letters within the same row are different (*P* < 0.05)

### Jejunal and ileal morphology

**Fig. 1:**
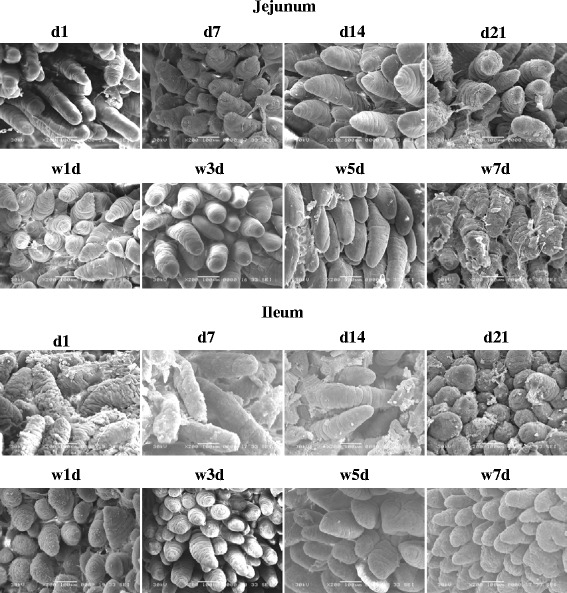
The representative images of scanning electron microscopy in the jejunum (A) and ileum (B) of 1, 7, 14, 21-d-old suckling piglets and piglets on days 1 (d 1 PW), 3 (d 3 PW), 5 (d 5 PW), 7 (d 7 PW) post-weaning at 14 d of age (magnification × 200, scale bar = 100 μm) (*n =* 8)

In suckling piglets, scanning electron microscopy (Fig. 1) showed that the villi in the jejunum on postnatal d 1 were long, thin and sparse, and then grew shorter, stouter and denser with age. The villi height in the ileum was increased on d 7 and then declined from d 7 to d 21. The results of intestinal hematoxylin-eosin staining showed that the ratio of villus height to crypt depth was highest on d 1 (*P* < 0.05) and then decreased with age whereas the number of goblet cells in the jejunum increased from d 1 to 21. In the ileum, villus height and the ratio of villus height to crypt depth decreased from 1 d to 21 d of age (Table 3) (*P* < 0.05).

**Table 3:** The jejunal and ileal morphology in the suckling and weaned piglets

Items	Day age				Day post-weaning				SEM	*P*-value
	1	7	14	21	1	3	5	7		
Jejunum										
Villus height, μm	397.50	386.75	282.93	295.70	305.55	263.13	261.60	320.63	15.04	0.143
Crypt depth, μm	171.13	220.35	248.63	195.93	151.78	202.33	166.20	194.55	17.31	0.924
Villus height : Crypt depth	2.32^a^	1.76^ab^	1.14^b^	1.51^b^	2.01^a^	1.30^b^	1.57^b^	1.65^b^	0.06	0.042
Goblet cell number	8.50^c^	11.50^b^	13.00^b^	20.75^a^	16.75^ab^	8.75^c^	16.75^ab^	14.50^b^	1.10	0.045
Lymphocyte number	99.25^b^	144.25^ab^	146.50^ab^	152.00^ab^	159.50^ab^	167.25^ab^	192.50^a^	151.25^ab^	1.76	0.037
Ileum										
Villus height, μm	428.58^a^	269.13^b^	273.53^b^	272.10^b^	260.80^b^	208.70^c^	243.50^bc^	267.05^b^	4.32	0.017
Crypt depth, μm	179.15	129.18	138.03	144.43	134.60	168.23	161.75	137.33	7.39	0.665
Villus height : Crypt depth	2.39^a^	2.08^ab^	1.98^b^	1.88^b^	1.94^b^	1.24^c^	1.51^c^	1.94^b^	0.04	0.041
Goblet cell number	27.25	29.00	22.25	33.00	19.50	24.75	23.50	33.00	1.41	0.129
Lymphocyte number	192.25	282.50	195.00	147.75	187.25	203.25	200.75	177.25	12.39	0.307

^1^The jejunum and ileum tissues were obtained from 1, 7, 14, 21-d-old suckling piglets and piglets on d 1, 3, 5, 7 post-weaning at 14 d of age

^2^ *n =* 8. ^a-c^Values with different letters within the same row are different (*P* < 0.05)

Weaning at 14 d induced a decrease in villi height and density, especially on d 3, but recovered on d7 post-weaning in both the jejunum and ileum (Fig. 1). Within the first week post-weaning after 14 d of age, the ratio of villus height to crypt depth and the goblet cell number were lowest on d 3, while the lymphocyte number was the greatest on d 5 post-weaning in the jejunum (*P* < 0.05). However, there were no significant differences in villus height, crypt depth or the numbers of goblet cells and lymphocytes between suckling and weanling piglets on d 21 in the jejunum. The lowest villus height and ratio of villus height to crypt depth were observed on d 3 and 5 post-weaning (*P* < 0.05) , but no differences were observed between suckling and weanling piglets on d 21 in the ileum (Table 3).

### PCNA immunohistochemical staining and alkaline phosphatase activity in the jejunum

**Fig. 2:**
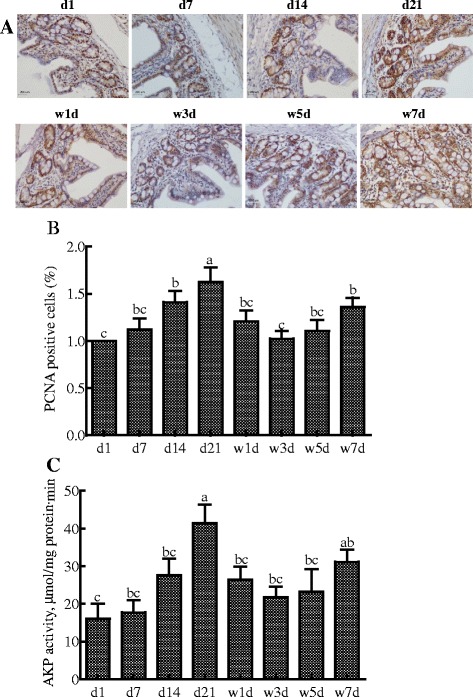
The representative images of immunohistochemical staining (magnification × 200,scale bar = 200 μm) (**a**), the percentage of PCNA positive cells (**b**) and the AKP activity (**c**) in jejunum of 1, 7, 14, 21-d-old suckling piglets and piglets on days 1 (d 1 PW), 3 (d 3 PW), 5 (d 5 PW), 7 (d 7 PW) post-weaning at 14 d of age. PCNA: Proliferating cell nuclear antigen; AKP: alkaline phosphatase. Data are expressed as means ± SEM, *n =* 8. ^a-c^Values with different letters are significantly different (*P* < 0.05)

Cell proliferation and differentiation of enterocytes were detected by monitoring PCNA labeling index and AKP activity [21, 22] that are shown in Fig. 2. The percentage of PCNA-positive cells and AKP activity in the jejunum increased from d 1 to 21 and reached the highest levels on d 21 in suckling piglets (*P* < 0.05).

Within the first week post-weaning after 14 d of age, the percentage of PCNA-positive cells in the jejunum was significantly decreased on d 3 (*P* < 0.05) , but recovered to the original level on d 7 post-weaning. There were no differences in AKP activity in the jejunum among 14-d-old suckling piglets and piglets on days 1, 3, 5, or 7 post-weaning, or between suckling and weanling piglets on d 21 (*P* > 0.05) (Fig. 2).

Relative mRNA abundances of E-cadherin, occludin, ZO-1, integrin, Kv1.1, and Kv1.5 in the jejunal and ileal mucosa of piglets

The relative mRNA abundances of E-cadherin, occludin, ZO-1, integrin, Kv1.1 and Kv1.5 in the intestinal mucosa of piglets are shown in Table 4. In suckling piglets, the mRNA abundances of E-cadherin, occludin and Kv1.5 tended to increase from d 1 to 21 , and rose to their highest levels on d 21 , whereas the Kv1.1 mRNA abundance in the jejunal mucosa was significantly decreased on d 21 (*P* < 0.05). The relative mRNA abundances of E-cadherin, ZO-1 and Kv1.5, and Kv1.1 in the ileal mucosa were the highest on d 21, 7, and 14, respectively, during d 1 to 21 (*P* < 0.05).

**Table 4:** The relative mRNA abundances of *E-cadherin*, *occludin*, *ZO-1*, *integrin, Kv1.1 and Kv1.5* in the jejunal and ileal mucosa of suckling and weaned piglets

Items	Day age				Day post-weaning				SEM	*P*-value
	1	7	14	21	1	3	5	7		
Jejunal mucosa										
*E-cadherin*	1.00^b^	1.42^b^	1.64^b^	2.79^a^	1.55^b^	0.38^c^	0.61^c^	1.10^bc^	0.056	<0.001
*occludin*	1.00^b^	0.62^b^	1.40^b^	5.00^a^	0.63^b^	0.32^c^	0.47^c^	0.79^b^	0.087	<0.001
*ZO-1*	1.00^b^	1.24^ab^	1.08^b^	1.30^a^	1.01^ab^	0.66^c^	0.86^bc^	0.99^b^	0.093	0.002
*integrin*	1.00^b^	1.08^ab^	1.13^ab^	1.48^a^	0.72^b^	0.62^b^	1.20^ab^	0.98^ab^	0.148	0.012
*Kv1.1*	1.00^b^	1.76^ab^	2.02^ab^	0.39^c^	1.89^ab^	2.48^a^	1.14^b^	1.17^b^	0.160	0.029
*Kv1.5*	1.00^b^	2.50^ab^	3.13^ab^	3.23^ab^	3.96^a^	4.76^a^	2.77^ab^	1.53^b^	0.235	<0.001
Ileal mucosa										
*E-cadherin*	1.00^b^	1.39^b^	1.50^b^	2.55^a^	0.97^bc^	0.34^c^	0.81^bc^	1.49^b^	0.081	<0.001
*occludin*	1.00^b^	1.15^b^	2.29^a^	3.52^a^	1.00^b^	0.87^b^	1.49^b^	1.78^b^	0.154	0.029
*ZO-1*	1.00^b^	1.80^a^	0.98^b^	0.86^b^	0.74^b^	0.65^b^	0.89^b^	0.90^b^	0.092	<0.001
*integrin*	1.00	1.23	0.81	0.99	0.83	0.49	0.65	0.49	0.186	0.251
*Kv1.1*	1.00^d^	3.32^b^	5.27^a^	2.36^bc^	4.39^ab^	2.42^bc^	1.84^c^	1.20^d^	0.206	0.001
*Kv1.5*	1.00^b^	2.77^a^	1.31^b^	0.95^b^	1.54^b^	1.41^b^	1.69^b^	1.25^b^	0.112	<0.001

*ZO-1* Zonula occludens, *Kv1.1* Potassium voltage-gated channel, shaker-related subfamily, member 1, *Kv1.5* Potassium voltage-gated channel, shaker-related subfamily, member 5

The jejunal and ileal mucosa were obtained from 1, 7, 14, 21-d-old suckling piglets and piglets on d 1, 3, 5, 7 post-weaning at 14 d of age

*n =* 8. ^a-d^Values with different letters within the same row are different (*P* < 0.05)

Within the first week post-weaning after 14 d of age, the abundances of E-cadherin mRNA on d 3 and 5 and ZO-1 mRNA on d 3 after weaning in the jejunal mucosa as well as E-cadherin and ZO-1 mRNA on d 3 and Kv1.1 mRNA on d 3, 5, and 7 in the ileal mucosa were significantly decreased compared with those on d 14 in suckling piglets (*P* < 0.05). In addition, there were lower abundances of E-cadherin, occludin and ZO-1 mRNA in the jejunal mucosa and of E-cadherin and Kv1.1 mRNA in the ileal mucosa in weanling piglets compared to suckling piglets at d 21 of age (*P* < 0.05) (Table 4).

Correlation analyses showed that the relative mRNA abundances of E-cadherin, occludin, ZO-1 and integrin were positively related to the percentage of PCNA-positive cells (r = 0.843, 0.803, 0.628 and 0.639, respectively) but no correlatively with AKP activity in the jejunal mucosa (Table 5).

**Table 5:** Correlation coefficients (r) between the percentage of PCNA positive cells and the relative mRNA abundances of *E-cadherin*, *occludin*, *ZO-1*, *integrin, Kv1.1 and Kv1.5* in the jejunal mucosa of piglets

Items		The relatvie mRNA abundances					
		*E-cadherin*	*Occludin*	*ZO-1*	*Integrin*	*Kv1.1*	*Kv1.5*
PCNA	Correlation coefficient	0.843^**^	0.803^**^	0.628^*^	0.639^*^	−0.458	0.045
	Sig.(1-tailed)	0.004	0.008	0.048	0.044	0.127	0.458
AKP	Correlation coefficient	0.246	0.217	−0.039	0.267	−0.175	−0.134
	Sig.(1-tailed)	0.099	0.148	0.788	0.070	0.223	0.362

*ZO-1* Zonula occludens, *Kv1.1* Potassium voltage-gated channel, shaker-related subfamily, member 1, *Kv1.5* Potassium voltage-gated channel, shaker-related subfamily, member 5; *PCNA* Proliferating cell nuclear antigen

Levels of significance: ^*^ *P* < 0.05; ^**^ *P* < 0.01

### Relative protein abundances of claudin-1, claudin-3, occludin and ZO-1 in the jejunum and ileum of piglets

In suckling piglets, the relative protein abundances of claudin-1, claudin-3, occludin and ZO-1 in the jejunum from d 1 to 21 tended to increase and reached their highest levels on d 21 (*P* < 0.05) (Table 6). The protein abundance of occludin on d 21 was greater (*P* < 0.05) than those at other ages, and there were no differences (*P* > 0.05) in the protein abundances of claudin-1, claudin-3 and ZO-1 in the ileum during the suckling period.

**Table 6:** The relative protein abundances of claudin-1, claudin-3, occludin and ZO-1 in the jejunum and ileum of suckling and weaned piglets

Items	Day age				Day post-weaning				SEM	*P*-value
	1	7	14	21	1	3	5	7		
Jejunum mucosa										
Claudin-1	1.00^c^	1.19^c^	1.31^c^	6.96^a^	0.89^c^	0.83^c^	2.03^bc^	4.52^b^	0.261	<0.001
Claudin-3	1.00^c^	1.93^c^	11.01^b^	25.91^a^	12.24^b^	2.76^c^	11.11^b^	13.62^b^	1.761	<0.001
Occludin	1.00^c^	2.12^b^	2.23^b^	3.10^a^	0.51^d^	1.50^c^	0.87^d^	2.57^ab^	0.147	<0.001
ZO-1	1.00^c^	0.83^c^	3.08^a^	3.90^a^	1.71^b^	1.33^bc^	0.55^c^	1.83^b^	0.237	<0.001
Ileum										
Claudin-1	1.00^b^	0.72^b^	0.79^b^	1.43^ab^	1.39^ab^	0.98^b^	2.19^a^	1.27^ab^	0.206	0.010
Claudin-3	1.00	0.89	1.08	1.38	1.02	0.67	0.72	1.26	0.175	0.134
Occludin	1.00^bc^	1.47^ab^	1.42^b^	1.93^a^	0.72^c^	0.86^c^	1.62^ab^	1.89^a^	0.142	<0.001
ZO-1	1.00^a^	0.88^ab^	1.05^a^	0.86^ab^	0.68^bc^	0.33^c^	0.42^bc^	1.05^a^	0.089	<0.001

*ZO-1* Zonula occludens

The jejunum and ileum were obtained from 1, 7, 14, 21-d-old suckling piglets and piglets on days 1, 3, 5, 7 post-weaning at 14 d of age

*n* = 8. ^a-d^Values with different letters within the same row are different (*P* < 0.05)

Compared with the values in 14-d-old suckling piglets, there were remarkable decreases in the relative protein abundances of claudin-3 on d 3, occludin on d 1, 3, and 5 and ZO-1 on d 1, 3, 5, and 7 after weaning in the jejunum (*P* < 0.05). Although there was some recovery on d 7 post-weaning, the protein abundances of claudin-1, claudin-3 and ZO-1 in the jejunum were still lower than those in 21-d-old suckling piglets (*P* < 0.05). Compared with the values in 14-d-old suckling piglets, there were remarkable decreases (*P* < 0.05) in the relative protein abundances of occludin and ZO-1 on d 1, 3 and 5 after weaning in the ileum, while there were no differences in the abundances of any of the proteins examined between suckling and weanling piglets on d 21 (Table 6).

## Discussion

The gastrointestinal tract of piglets is exposed to a wide array of stress factors during the early postnatal and post-weaning periods, which are characterized by significant growth and morphological changes [23]. The small intestine grows more rapidly than the body as a whole both before and after birth in piglets [24], and has a 50 % higher relative weight at 24 h after birth than at birth [3]. Intestinal crypt depth increases by 40 % and villi height increases by 35 % [23]. The microvilli of the jejunum on postnatal d 1 were long, thin and sparse, and then grew shorter, stouter and denser with age in the present study, which was associated with an increase in the goblet cell number, which indicates an increase in the absorption area of the intestinal mucosa. Marion et al. [25] demonstrated that the absorption area increased by about 50 % on the first postnatal day and by 100 % during the first 10 postnatal days. A large luminal surface area with optimal enterocyte functional maturity is important for young growing pigs so that they may attain their maximum digestive and absorptive capability.

The intestine of neonatal piglets is also regarded to be anatomically and functionally immature and is hypersensitive to weaning. Weaning stress in pigs has been reported to impair the intestinal architecture and function, which leads to gut-associated disorders and diarrhea [6, 26–28]. The intestinal mucosa will show a 20 ~ 30 % reduction in relative weight during the first 2 d post-weaning, and will need 5 ~ 10 d for full recovery [29]. The villus height has a profound effect on the intestinal structure, and may be reduced to 75 % of the pre-weaning values within 24 h of weaning at 21 d of age [30]. In the current study, weaning at 14 d of age induced a decrease in the ratio of villus height to crypt depth in the jejunum and ileum on d 3, 5 post-weaning, which is in agreement with a report that the most serious damage to the intestinal barrier occurred from d 3 to d 5 post-weaning [6, 31]. Along with the reduction in villus height and the increase in crypt depth, the specific activity of brush-border enzymes has also been shown to decrease to minimum values during d 4 to 5 after weaning [32, 33]. The mucosa of the intestinal tract has the unique ability to repair itself rapidly after damage [34]. Hu et al. [6] suggested that villus height and crypt depth returned to pre-weaning values on d 14 post-weaning. In the present study, there were also no significant differences in villus height or crypt depth between suckling and weanling piglets on d 21.

Intestinal and mucosal development is driven by accelerated epithelial cell proliferation [35, 36], and enhanced proliferation in the crypt area is associated with structural and functional remodeling of the epithelium [37, 38]. PCNA is a 36 kD nuclear protein that is required for DNA synthesis and repair, and is closely associated with DNA polymerase in the S-phase of the cell cycle [39]. The jejunal PCNA labeling index gradually increased in association with the mucosal maturation from d 1 to 21 in suckling piglets. However, it has been shown that weaning stress induced cell cycle arrest, enhanced apoptosis, and inhibited cell proliferation [40]. In the present study, a sharp decrease was observed in the percentage of PCNA-positive cells on 3 d post-weaning.

The mucosal restitution process also involves cell differentiation and migration [37, 38]. The rapid mucosal restitution of superficial wounds in vivo is function due to differentiated intestinal epithelial cells from the surface of the mucosa rather than undifferentiated epithelial cells within the crypts [10]. AKP activity, a marker of cell differentiation [22, 41], increased progressively from postnatal d 1 to 21, which may be explained by the fact that brush border enzymes are glycoproteins for which activity increases with age. However, weaning decreased the maximal AKP enzyme activity and AKP gene expression in the small intestine [42]. In this study, these tended to decrease on d 3 post-weaning (Fig. 2). A gradual increase in the abundance of Kv1.5 mRNA from d 1 to 21 in suckling piglets and a trending increase at 3 d post-weaning in the jejunal mucosa were also observed. K^+^ channel activation has been associated with growth or differentiation in many cells [43]. An increase in Kv channel expression indicated that it plays a critical role in the process of differentiated intestinal epithelial cell migration after wounding [10], which has been demonstrated in the regulatory effect of polyamine on mucosal restitution in a previous study [17].

It has also been demonstrated that weaning of piglets negatively affected small intestinal integrity as indicated by an increase in paracellular permeability [6]. Here, plasma D-lactate and DAO, two well-established markers, were used to estimate intestinal permeability. Increasing in the levels of plasma diamine oxidase and D-lactate was accompanied with injure of intestinal epithelial cells [7, 44]. Only an increase in the D-lactate concentration on d 1 post-weaning was observed and there were no differences in DAO content with 7 days post-weaning. These results did not entirely chime with the intercellular junctional proteins, an important component of the intestinal barrier, which is crucial for the maintenance of barrier integrity [45]. The abundances of E-cadherin and occludin mRNA as well as occludin and ZO-1 protein in the jejunum and ileum were decreased on d 3 and 5 post-weaning, which indicated that weaning impaired the intestinal barrier. These results were consistent with the reports of Hu et al. [6] and Xiao et al. [7], who demonstrated that tight junction protein levels were decreased on d 3 and/or d 7 post-weaning. The stress of heat and oxidative damage also disrupted intestinal cell tight junction proteins, resulting in increased permeability to luminal endotoxins [46]. The differences between suckling and weanling piglets at days 15, 17, 19 and 21 of age were not investigated in the present study. Although it is not known whether the above changes in morphology and related genes and proteins were due to age or weaning, weaning should play an important part in the damage to the intestinal morphology and barrier. In this study, the abundances of E-cadherin, occludin and ZO-1 mRNA as well as claudin-1, claudin-3 and ZO-1 protein in the jejunum of weanling piglets were lower than those in suckling piglets at age 21 d.

## Conclusion

The present results indicated a progressive increase in cell proliferation, AKP activity and intercellular junction protein expression in the small intestine of piglets with age during the suckling period from d 1 to 21. Weaning at d14 of age induced an increase in the plasma D-lactate content and intestinal Kv channel expression, but a decrease in villus height/crypt depth, cell proliferation, AKP activity and the expression of intercellular junction proteins in the small intestine. The most serious damage to the intestinal morphology and barrier occurs on d 3 or 5. While there is an adaptive restoration on d 7 post-weaning, the measured values do not return to the pre-weaning levels. These results should help to improve the adaptation to weaning in piglets. The promotion of intestinal maturation and epithelial restitution may help to alleviate the adverse effects of weaning.

## References

[1] Catalioto R, Maggi C, Giuliani S (2011). Intestinal epithelial barrier dysfunction in disease and possible therapeutical interventions. Curr Med Chem.

[2] Gao JH, Guo LJ, Huang ZY, RAO JN, Tang CW (2013). Roles of cellular polyamines in mucosal healing in the gastrointestinal tract. J Physiol Pharmacol.

[3] Adeola O, King DE (2006). Developmental changes in morphometry of the small intestine and jejunal sucrase activity during the first nine weeks of postnatal growth in pigs. J Anim Sci.

[4] Montagne L, Boudry G, Favier C, Le Huërou-Luron I, Lallès JP, Sève B (2007). Main intestinal markers associated with the changes in gut architecture and function in piglets after weaning. Br J Nutr.

[5] Campbell J, Crenshaw J, Polo J (2013). The biological stress of early weaned piglets. J Anim Sci Biotechnol.

[6] Hu CH, Xiao K, Luan ZS, Song J (2013). Early weaning increases intestinal permeability, alters expression of cytokine and tight junction proteins, and activates mitogen-activated protein kinases in pigs. J Anim Sci.

[7] Xiao K, Song ZH, Jiao LF, Ke YL, Hu CH (2014). Developmental Changes of TGF-β1 and Smads Signaling Pathway in Intestinal Adaption of Weaned Pigs. PLoS One.

[8] Dignass AU (2001). Mechanisms and modulation of intestinal epithelial repair. Inflamm Bowel Dis.

[9] Blikslager AT, Moeser AJ, Gookin JL, Jones SL, Odle J (2007). Restoration of barrier function in injured intestinal mucosa. Physiol Rev.

[10] Rao JN, Platoshyn O, Li L, Guo X, Golovina VA, Yuan JX (2001). Activation of K^+^ channels and increased migration of differentiated intestinal epithelial cells after wounding. Am J Physiol Cell Physiol.

[11] Cereijido M, Shoshani L, Contreras R (2000). Molecular physiology and pathophysiolgy of tight junctions. I. Biogenesis of tight junctions and epithelial polarity. Am J Physiol Gastrointest Liver Physiol.

[12] Liu Y, Nusrat A, Schnell F, Reaves T, Walsh S, Pochet M (2000). Human junction adhesion molecule regulates tight junction resealing in epithelia. J Cell Sci.

[13] Van Itallie CM, Anderson JM (2014). Architecture of tight junctions and principles of molecular composition. Semin Cell Dev Biol.

[14] Yin J, Wu MM, Xiao H, Ren WK, Duan JL, Yang G (2013). Development of an antioxidant system after early weaning in piglets. J Anim Sci.

[15] Xiao H, Tan BE, Wu MM, Yin YL, Li TJ, Yuan DX (2013). Effects of composite antimicrobial peptides in weanling piglets challenged with deoxynivalenol: II. Intestinal morphology and function. J Anim Sci.

[16] German DP (2009). Inside the guts of wood-eating catfishes: can they digest wood?. J Comp Physiol B.

[17] Liu W, Shan LP, Dong XS, Liu XW, Ma T, Liu Z (2013). Combined early fluid resuscitation and hydrogen inhalation attenuates lung and intestine injury. World J Gastroenterol.

[18] Wang J, Li GR, Tan BE, Xion X, Kong XF, Xiao DF (2015). Oral administration of putrescine and proline during the suckling period improves epithelial restitution after early weaning in piglets. J Anim Sci.

[19] Tan BE, Li XG, Kong XF, Huang RL, Ruan Z, Yao K (2009). Dietary L-arginine supplementation enhances the immune status in early-weaned piglets. Amino Acids.

[20] Tan BE, Yin YL, Liu ZQ, Tang WJ, Xu HJ, Kong XF (2011). Dietary arginine supplementation differentially regulates expression of lipid-metabolic genes in porcine adipose tissue and skeletal muscle. J Nutri Biochem.

[21] Marcos MA, Vila J, Gratacos J, Brancos MA, de Anta MT J (1991). Determination of D-lactate concentration for rapid diagnosis of bacterial infections of body fluids. Eur J Clin Microbiol Infect Dis.

[22] Dezfuli BS, Giari L, Lui A, Squerzanti S, Castaldelli G, Shinn AP, Manera M, and Lorenzoni M. Parasit Vectors. Proliferative cell nuclear antigen (PCNA) expression in the intestine of Salmo trutta truttanaturally infected with an acanthocephalan. 2012;5:198. doi:10.1186/1756-3305-5-198 10.1186/1756-3305-5-198 PMC3583471 22967751

[23] Godlewski MM, Slupecka M, Wolinski J, Skrzypek T, Skrzypek H, Motyl T (2005). Into the unknown - the death pathways in the neonatal gut epithelium. J Physiol Pharmacol.

[24] Cheng H, Leblond CP (1974). Origin, differentiation and renewal of the four main epithelial cell types in the mouse small intestine. I Columnar cells Am J Anat.

[25] Marion J, Rome V, Savary G, Thomas F, Le Dividich J, Le Huerou-Luron I (2003). Weaning and feed intake alter pancreatic enzyme activities and corresponding mRNA levels in 7-d-old piglets. J Nutr.

[26] Wang J, Chen L, Li P, Li X, Zhou H, Wang F (2008). Gene expression is altered in piglet small intestine by weaning and dietary glutamine supplementation. J Nutr.

[27] Wijtten PJA, Meulen JVD, Verstegen MWA (2011). Intestinal barrier function and absorption in pigs after weaning: a review. Br J Nutr.

[28] McLamb BL, Gibson AJ, Overman EL, Stahl C, Moeser AJ (2013). Early weaning stress in pigs impairs innate mucosal immune responses to enterotoxigenic E. coli challenge and exacerbates intestinal injury and clinical disease. PLoS One.

[29] Lallès JP, Boudry G, Favier C, Le Floc’h N, Luron I, Montagne L (2004). Gut function and dysfunction in young pigs: physiology. Anim Res.

[30] Hampson DJ (1986). Alterations in piglet small intestinal structure at weaning. Res Vet Sci.

[31] Mei J, Xu R (2005). Transient changes of transforming growth factor-β expression in the small intestine of the pig in association with weaning. Br J Nutr.

[32] Tang M, Laarveld B, VanKessel AG, Hamilton DL, Estrada A, Patience JF (1999). Effect of segregated early weaning on postweaning small intestinal development in pigs. J Anim Sci.

[33] Fan MZ, Adeola O, Asem EK, King D (2002). Postnatal ontogeny of kinetics of porcine jejunal brush border membrane-bound alkaline phosphatase, aminopeptidase N and sucrase activities. Comp Biochem Physiol.

[34] Sangild PT, Fowden AL, Trahair JF (2000). How does the foetal gastrointestinal tract develop in preparation for enteral nutrition after birth?. Livest Prod Sci.

[35] Tan BE, Yin YL, Kong XF, Li P, Li XL, Gao HJ (2010). L-Arginine stimulates proliferation and prevents endotoxin-induced death of intestinal cells. Amino Acids.

[36] Tan BE, Xiao H, Xiong X, Wang J, Li GR, Yin YL (2015). L-Arginine improves DNA synthesis in LPS-challenged enterocytes. Front Biosci.

[37] de Santa BP, van den Brink GR, Roberts DJ (2003). Development and differentiation of the intestinal epithelium. Cell Mol Life Sci.

[38] Noah TK, Donahue B, Shroyer NF (2011). Intestinal development and differentiation. Exp Cell Res.

[39] Sanden M, Olsvik PA (2009). Intestinal cellular localization of PCNA protein and CYP1A mRNA in Atlantic salmon Salmo salar L. exposed to a model toxicant. BMC Physiol.

[40] Zhu L, Xu J, Zhu S, Cai X, Yang S, Chen X (2014). Gene expression profiling analysis reveals weaning-induced cell cycle arrest and apoptosis in the small intestine of pigs. J Anim Sci.

[41] Sabater-Molina M, Larque E, Torrella F, Plaza J, Lozano T, Munoz A (2009). Effects of dietary polyamines at physiologic doses in early-weaned piglets. Nutrition.

[42] Lackeyram D, Yang C, Archbold T, Swanson KC, Fan MZ (2010). Early weaning reduces small intestinal alkaline phosphatase expression in pigs. J Nutr.

[43] Urrego D, Tomczak AP, Zahed F, Stühmer W, Pardo LA (2014). Potassium channels in cell cycle and cell proliferation. Philos Trans R Soc Lond B Biol Sci.

[44] Hu CH, Gu LY, Luan ZS, Song J, Zhu K (2012). Effects of montmorillonite-zincoxide hybrid on performance, diarrhea, intestinal permeability and morphology of weanling pigs. Anim Feed Sci Tech.

[45] Ulluwishewa D, Anderson RC, McNabb WC, Moughan PJ, Wells JM, Roy NC (2011). Regulation of tight junction permeability by intestinal bacteria and dietary components. J Nutr.

[46] Zuhl M, Schneider S, Lanphere K, Conn C, Dokladny K, Moseley P (2014). Exercise regulation of intestinal tight junction proteins. Br J Sports Med.

